# COVID-19: FAQs in Pediatric Cardiac Surgery—A Sequel

**DOI:** 10.1177/2150135120953411

**Published:** 2020-09-10

**Authors:** Emily Levy, Jennifer Blumenthal, Kathleen Chiotos, Elizabeth H. Stephens, Joseph A. Dearani

**Affiliations:** 1 Divisions of Pediatric Infectious Diseases and Pediatric Critical Care Medicine, Department of Pediatric and Adolescent Medicine, 6915 Mayo Clinic, Rochester, MN, USA; 2 Division of Critical Care Medicine, Department of Anesthesiology, Critical Care and Pain Medicine, 1862 Boston Children’s Hospital, Boston, MA, USA; 3 Division of Infectious Diseases, Department of Medicine, 1862 Boston Children’s Hospital, Boston, MA, USA; 4 Divisions of Infectious Diseases and Critical Care Medicine, 6567 Children’s Hospital of Philadelphia, PA, USA; 5 Department of Cardiovascular Surgery, 6915 Mayo Clinic, Rochester, MN, USA

**Keywords:** COVID-19, congenital heart disease, pandemic, congenital heart surgery

## Introduction

As the world and the health care industry continue to adapt to the ever-changing COVID-19 pandemic, so too does our understanding of the virus, its detection, spread, and possible treatments evolve. While congenital cardiac surgery care resumes during this time, important issues centering on the infectious disease and critical care aspects of the COVID-19 pandemic remain at the forefront. These issues and questions related to them are central to our ability to provide care for our patients.^1^ As a sequel to our previous review, the purpose of this review is to succinctly summarize our current understanding of frequently asked questions regarding COVID-19 with respect to congenital heart disease.^2^ Some of these questions were included in the previous review, but updated information has become available. Meanwhile, additional questions have become relevant subsequent to the previous review. Our knowledge of the COVID-19 pandemic continues to grow rapidly, leading to further clarity and revealing new questions in turn, thus it is critically important that clinician knowledge is updated frequently.

1. At this point, what treatments are available for COVID-19? (updated)

The cornerstone therapy for COVID-19 is supportive care and, when appropriate, critical care. Critically ill children with refractory hypoxemia should be cared for in a center with dedicated specialists in pediatric critical care and the potential for extracorporeal membrane oxygenation (ECMO) support.^3^

Newer evidence has emerged suggesting that dexamethasone may reduce mortality when administered to adults with severe COVID-19 (eg, those requiring supplemental oxygen, invasive or noninvasive mechanical ventilation).^4^ Based on these data, both the Infectious Diseases Society of America and National Institutes of Health guidelines for the treatment of COVID-19 now recommend steroids for severe disease.^5^ It is unclear how to apply these data to children, given the overall more favorable prognosis of COVID-19 in the pediatric population; the authors suggest steroid use be considered on a case-by-case basis, particularly for children requiring mechanical ventilation.

https://www.medrxiv.org/content/10.1101/2020.06.22.20137273v1.full.pdf
https://journals.lww.com/ccmjournal/Fulltext/2020/06000/Surviving_Sepsis_Campaign__Guidelines_on_the.29.aspx
https://www.idsociety.org/practice-guideline/covid-19-guideline-treatment-and-management/

Remdesivir is an antiviral medication that appears to shorten time to clinical recovery in COVID-19 patients based on preliminary data from a single randomized trial performed in hospitalized adults. These data led to emergency use authorization by the US Food and Drug Administration (FDA) for use of remdesivir in neonates, infants, children, and adults requiring supplemental oxygen, invasive or noninvasive mechanical ventilation, or ECMO. Remdesivir should be considered for all pediatric patients meeting these criteria.^6^ In addition, the pharmacokinetics, safety, and efficacy of remdesivir is being studied in a pediatric clinical trial that is now enrolling at participating sites.

https://academic.oup.com/jpids/article/doi/10.1093/jpids/piaa045/5823622
https://www.fda.gov/news-events/press-announcements/coronavirus-covid-19-update-fda-issues-emergency-use-authorization-potential-covid-19-treatment

Hydroxychloroquine and chloroquine have been FDA approved for treatment and prophylaxis of uncomplicated malaria as well as many autoinflammatory conditions. Despite early enthusiasm for possible antiviral activity, multiple randomized trials and large observational studies have demonstrated that neither drug is effective in treating COVID-19 and may be associated with harm, including arrhythmias and QTc prolongation. Neither drug should be used for the treatment of COVID-19 outside a clinical trial.^5^

https://www.idsociety.org/practice-guideline/covid-19-guideline-treatment-and-management/

There is some data regarding convalescent plasma (transfused plasma from patients who have had COVID-19 and now have antibodies) in critically ill adults with COVID-19. In early application, this therapy appears to be safe, but efficacy has not yet been established.^7–9^ There may also be a role for use of convalescent plasma during pediatric critical illness, but analyses are in progress, and there are little data to date.

https://www.ncbi.nlm.nih.gov/pmc/articles/PMC7270883/
https://www.jci.org/articles/view/140200
https://www.ncbi.nlm.nih.gov/pmc/articles/PMC7368917/

2. What factors determine the optimal timing of surgery in patients who have been SARS-CoV-2 positive? (updated)

There remains very little evidence to suggest the optimal timing of surgery in COVID-19 positive patients. Choices should be individualized, considering both patient and provider safety. In general, a symptom-based strategy is recommended by both the Centers for Disease Control and Prevention (CDC) and the authors. When symptom-based clearance is very difficult (for instance, when the patient is asymptomatic, which appears more common in children as compared to adults), a polymerase chain reaction (PCR) test-based strategy may be considered. There are little data to support serology (immunoglobulin G [IgG])-based strategies. Some programs are using a time-based strategy such as a six-week period post infection. While this may be reasonable for elective cases, it is the authors’ opinion that the length of time may not be necessary, particularly for cases with some urgency. While we agree that anesthetic risk should be minimized by allowing adequate time to pass for recovery from lower respiratory tract disease, as would be done with many other viral respiratory infections, these decisions should be individualized. Shorter durations may be appropriate for more urgent cases and for patients with fewer (or absent) lower respiratory symptoms. This may be particularly true for congenital or perinatal positive PCRs. As always, clinical judgment on urgent or emergent need for procedure requires diligence on the part of the cardiothoracic surgeon and cardiologist.^1,10^

https://www.cdc.gov/coronavirus/2019-ncov/hcp/disposition-hospitalized-patients.html
https://www.ncbi.nlm.nih.gov/pmc/articles/PMC7307004/
https://pubmed.ncbi.nlm.nih.gov/32662334/

3. Are patients with positive SARS-CoV-2 IgG “protected?” What does it mean to have positive IgG serology?

Although there may be false positive IgG tests, particularly the rapid “bloodspot” IgG, in general, a true positive IgG implies prior infection with SARS-CoV-2. Immunoglobulin G antibodies typically rise 10 to 14 days after the initial infection. There is little evidence to date about the duration and strength of protective titers (“durable immunity”), though this is an area of active investigation. It is important to remember, however, that clinicians should continue to utilize appropriate personal protective equipment (PPE) when working, and observe social distancing and masking recommendations, regardless of serologic status.

4. Is the virus “mutating?” What does this mean clinically?

SARS-CoV-2 is a beta coronavirus. The coronavirus family is a class of enveloped, positive-sense single-stranded RNA viruses. In general, RNA viruses are less stable than DNA viruses and tend to develop point mutations over time as they spread throughout populations due to less precise transcription. Selective pressure may encourage certain “strains” or subtypes. Recent population genetic analysis indicates at least 2 major lineages that are well defined, as well as multiple other point mutations.^11^ The significance of these strain subtypes with regard to virulence and transmissibility remains unclear. There is no evidence to date that humans could have repeated infections from different strains of SARS-CoV-2, but there are very little data at this point.

https://academic.oup.com/nsr/article/7/6/1012/5775463
https://www.mdpi.com/675796

5. What is MIS-C? How is it related to Kawasaki disease? Does MIS-C cause coronary aneurysms?

Multisystem inflammatory syndrome in children (MIS-C) is hypothesized to be a postinfectious hyperinflammatory syndrome in children triggered by SARS-CoV-2. It remains rare. Multiple organ systems are involved, including the cardiovascular system, and a large percentage of children with MIS-C have hypotension, vasodilation, and/or ventricular dysfunction. Some studies have estimated more than 50% of children with MIS-C require intensive care unit (ICU), primarily for hemodynamic support. Patients may also have gastrointestinal involvement, hypercoagulation, respiratory distress, mucocutaneous involvement, neurologic symptoms, and a variety of other inflammation-related organ dysfunction.^12,13^

The case definition from the CDC includes any individual <21 years with

- fever,
- laboratory evidence of inflammation,
- 2 or more organ systems involved,
- lab evidence of COVID-19 or COVID-19 exposure, and
- exclusion of other plausible diagnoses.

Initially, parallels were drawn to Kawasaki disease (KD) because some of the symptoms, including conjunctival injection, rash, and fever, were similar. However, as more data have been published, major differences have emerged. In comparison to KD presentations, MIS-C patients tend to be older, have prominent gastrointestingal symptoms, are much more likely to present in shock, tend to present with lymphopenia, and have higher inflammatory markers. Children with MIS-C may develop coronary artery aneurysms and require long-term echocardiogram surveillance and, in some cases, antiplatelet therapy.^12,13^ There are no data at this point about the long-term outcomes of patients with MIS-C.

https://www.cdc.gov/mis-c/
https://www.ncbi.nlm.nih.gov/pmc/articles/PMC7346765/
https://pubmed.ncbi.nlm.nih.gov/32653054/

6. How is MIS-C managed and treated?

Unlike children with acute COVID-19 infection, MIS-C patients tend to have less respiratory distress/failure and more prominent hemodynamic compromise. However, excellent supportive and critical care are also the cornerstones of management for this syndrome—particularly with regard to early recognition and aggressive management to reverse shock. Potential therapies for MIS-C include intravenous immunoglobulin (most commonly used), steroids (variable doses reported), and antiplatelet therapy, particularly in patients who also meet KD criteria and in those with coronary artery aneurysms. Some centers also treat with immunomodulators, which may include anti-interleukin-6 (IL6) (tocilizumab) or anti-IL-1Ra (anakinra) therapy.^12,13^ There is no evidence to date about the most effective treatments although several observational studies and registries are underway.

https://www.cdc.gov/mis-c/
https://www.ncbi.nlm.nih.gov/pmc/articles/PMC7346765/
https://pubmed.ncbi.nlm.nih.gov/32653054/

7. What are the current travel recommendations regarding COVID-19 exposure?

At this time, COVID-19 incidence is still quite high in many regions in the United States. Following local public health guidance regarding social distancing and masking is the best way to protect yourself from exposure. If travel is required, it may be safer to avoid air travel for shorter distances that are achievable by private vehicle. However, there is no evidence at this time that demonstrates that air travel is significantly higher risk than cross-state car travel that requires stops at gas stations or other public places. Most respiratory viruses *do not* spread easily on flights because of strict airflow and filter requirements on airplanes. However, social distancing may be more difficult on flights. If you must travel, or if your patient must travel to obtain appropriate medical and surgical care, we recommend following CDC and local government guidelines including hand washing, avoiding touching your face, and wearing a face covering such as a simple surgical mask.

https://www.cdc.gov/coronavirus/2019-ncov/travelers/travel-in-the-us.html

8. Will there be a vaccine soon for COVID-19?

By early August 2020, five vaccine candidates had entered or were about to embark upon phase 3 clinical trials in the United States. If a candidate is found to be both effective and safe, experts speculate a vaccine may be widely available within the next 6 to 12 months. It is critical to remember that this accelerated time line is made possible by unprecedented partnerships (eg, financial investment) between the federal government and private industry—and *not* by a loosening of the established regulatory standards for vaccine safety. These regulatory standards require the completion of large phase 3 trials establishing safety and efficacy prior to vaccine licensure by the FDA.

More specifically, federal investment has facilitated phase 3 trials beginning now, whereas pharmaceutical companies would otherwise require additional promising early phase data supporting vaccine immunogenicity prior to taking the financial risk of funding a costly phase 3 efficacy trial involving tens of thousands of people. Additionally, the federal government has invested in upscaling vaccine production, even before efficacy data are available, although these vaccines will only be distributed if approved. While both of these maneuvers will shorten the time until an effective vaccine is available, the critical step for establishing safety and efficacy—appropriately conducted phase 3 trials—is not, and cannot be, accelerated.^14,15^

https://www.nih.gov/research-training/medical-research-initiatives/activ
https://pubmed.ncbi.nlm.nih.gov/32628244/
https://academic.oup.com/jpids/article/doi/10.1093/jpids/piaa093/5879970

9. What PPE and which masks are appropriate for health care workers to wear for protection against COVID-19?

During regular clinical care encounters involving patients with COVID-19, health care workers should wear surgical face masks, eye protection, gowns, and gloves, in both inpatient and outpatient settings.^16,17^

https://academic.oup.com/jpids/article/doi/10.1093/jpids/piaa082/5875945
https://pubmed.ncbi.nlm.nih.gov/32497510/

10. Isn’t the virus “airborne?” When do I need an N95/powered air-purifying respirator?

Much debate surrounds the mode of transmission of SARS-CoV-2 and whether the transmission is primarily by droplets or aerosols (“airborne”). Respiratory droplets are larger particles (>5 µm) that fall to the ground within 3 to 6 ft of the individual producing them, whereas aerosols are smaller and lighter, qualities that allow them to stay suspended in the air and allow transmission without close physical proximity. Infections with droplet spread may be protected against with eye protection and simple surgical masks. Infections that have airborne transmission (like tuberculosis or measles) remain in the air as smaller aerosols and require N95/powered air-purifying respirator (PAPR) protection.^17^

It is important for clinicians to recognize that the droplet versus airborne classification is actually nonbinary and exists on a spectrum. So, it is the goal of epidemiologic inquiries to establish the *predominant* mode of transmission in the “real-world” clinical setting. While studies performed under experimental conditions have demonstrated the presence of SARS-CoV-2 aerosols, the detection of aerosols does not necessarily equate to “real-world” aerosol-based transmission. The observed characteristics of SARS-CoV-2 transmission in general populations, among household contacts, and among health care workers are most consistent with primarily droplet spread.^18^ That said, it is prudent for health care workers to don N95s or PAPRs during procedures in which aerosols are generated in higher quantity (eg, intubation, bronchoscopy) often grouped under the nomenclature “aerosol generating procedures.” Regardless of mask type, staff should wear eye protection, gowns, and gloves when caring for patients with COVID-19. The authors recommend compliance with local Infection Control guidelines; these may evolve over time based on local epidemiology and as additional data become available.

https://www.cdc.gov/coronavirus/2019-ncov/hcp/infection-control.html
https://pubmed.ncbi.nlm.nih.gov/32497510/
https://jamanetwork.com/journals/jama/fullarticle/2768396

## Summary

COVID-19 is not going away anytime soon and it continues to pose unprecedented challenges to the world and to the health care industry, particularly the cardiac surgical specialties where exposure is potentially greater because of disease complexity, longer ICU durations, and overall hospital length of stay. This ongoing ordeal has precipitated unmatched collaborations among congenital heart surgeons across regions, nations, and continents, as well as unifying partnerships with other specialties devoted to the care of these complex patients. This partnership among cardiac surgeons, intensivists, and infectious disease colleagues is an example of the synergy we can develop to continue to improve care for our complex patients in this new era. Unprecedented times in this crucible of trial necessitate standardization, constant reevaluation of new information, and instant readiness and adaptability in order to safely improve the care of our patients. That’s what we are doing. As the pandemic and virus evolve, we will persist in the examination of COVID-19-related information and how it impacts our patients, so we provide the best care during these ever-changing times.

## Abbreviations and Acronyms

CDC: Centers for Disease Control and Prevention
ECMO: extracorporeal membrane oxygenation
FDA: Food and Drug Administration
ICU: intensive care unit
IgG: immunoglobulin G
IL: interleukin-6
KD: Kawasaki disease
MIS-C: multisystem inflammatory syndrome in children
PAPR: powered air-purifying respirator
PCR: polymerase chain reaction
PPE: personal protective equipment
